# 1852. Harnessing the Power of Artificial Intelligence to Mitigate the HIV/STI Epidemic: Identifying Free Resources for Prevention and Treatment

**DOI:** 10.1093/ofid/ofad500.1680

**Published:** 2023-11-27

**Authors:** Premalkumar M Patel, Freddie Prieto, Yavani Singh, Claudio Tuda, Cynthia Rivera

**Affiliations:** Mount Sinai Medical Center of Florida, Hollywood, Florida; Mount Sinai Medical Center, Miami Beach, Florida; Mount Sinai Medical Center, Miami Beach, Florida; Mount Sinai Medical Center, Miami Beach, Florida; Mount Sinai Medical Center, Miami Beach, Florida

## Abstract

**Background:**

According to the CDC, 1.2 million people in the US have contracted HIV, with 13% of them being unaware of their diagnosis and treatment options. Only 25% of the eligible population is using PrEP. At any given time, 1 in 5 people in the US have STIs, but many do not seek medical attention due to the associated stigma and discrimination. Advanced technologies, such as AI, are being utilized to enhance healthcare delivery and address disparities in accessing health services. AI algorithms can help reduce the spread of HIV and STIs and prevent long-term complications.

HIV/STI free clinic information for providers/patients

**Figure:**
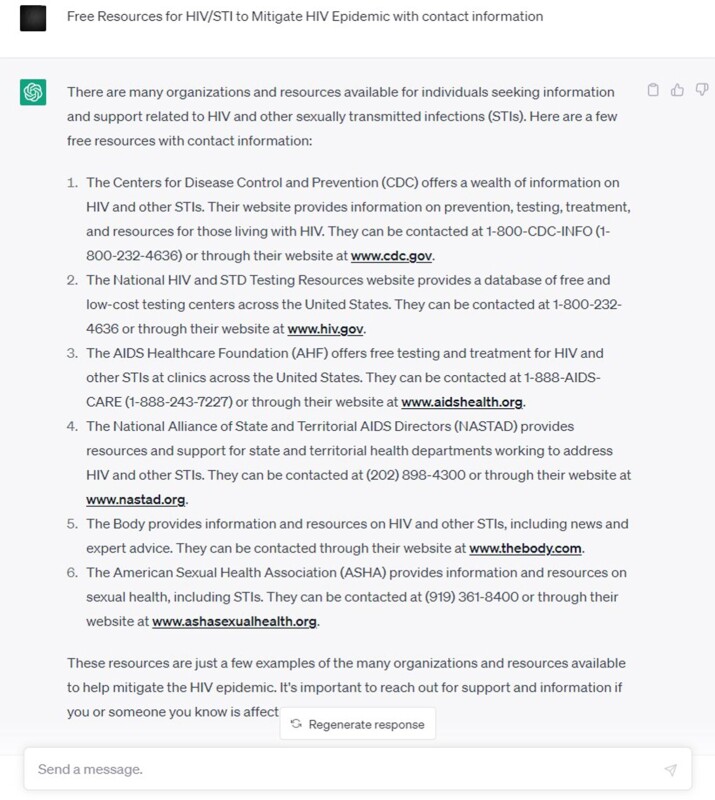

HIV/STI free resources information generated by ChatGPT

**Figure:**
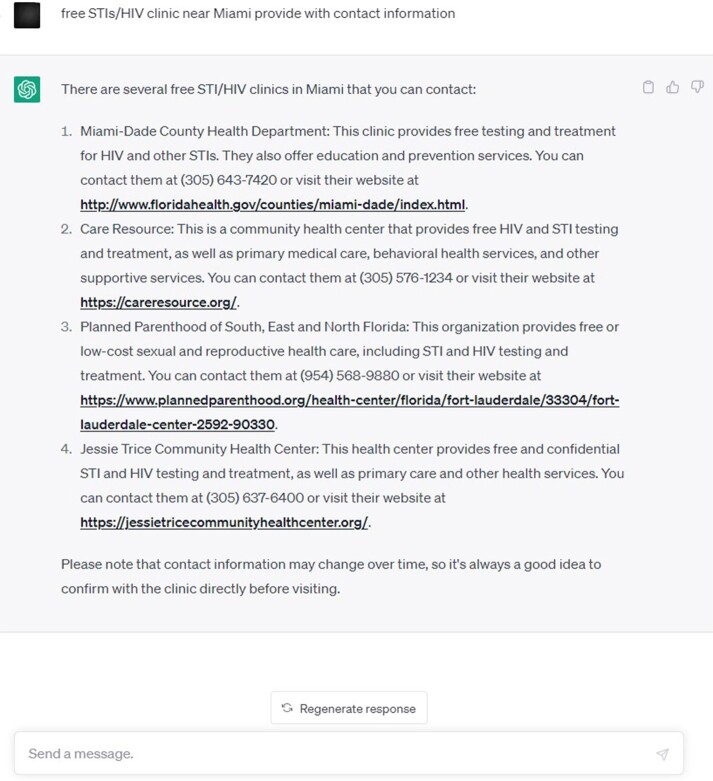

HIV/STI free clinic information near Miami generated by ChatGPT

**Methods:**

To access ChatBot in ChatGPT for finding resources, we followed these steps:

1. Open a web browser and go to the ChatGPT website.
2. On the homepage, select the "Academic" option.
3. Once the academic chatbot window loads, started by typing our question, "Free HIV/STI testing and treatment resources."
4. The ChatBot provided a response to our question.
5. If the response requires further clarification, the ChatBot will prompt you to provide more details.

**Results:**

The Chatbot was utilized by the ChatGPT application and conducted an extensive search for HIV/STI testing and treatment resources in the US. The search involved identifying potential resources from numerous sources, such as Planned Parenthood clinics, community health centers, care resources, AHF and the National HIV and STD Testing Resources websites. The search was focused on determining whether insurance was required, the type of services provided, and contact information for the potential resources. Screenshots attached as images.

**Conclusion:**

In conclusion, the utilization of artificial intelligence in finding free resources for HIV/STI prevention and treatment can be a game-changer in addressing the HIV epidemic. With its ability to identify available resources and high-risk populations, AI can greatly aid in allocating resources more efficiently towards reducing barriers that impede access to HIV/STI prevention and treatment.Further research is needed to explore how best AI can enhance these findings but undoubtedly it could be valuable if applied correctly. Overall, the implementation of AI-based solutions may hold significant promise for addressing public health challenges posed by STIs/HIV infections on a global scale.

**Disclosures:**

**All Authors**: No reported disclosures

